# Microsatellite instability/mismatch repair deficiency and activation of the Wnt/β-catenin signaling pathway in gastric adenocarcinoma of the fundic gland: A case report

**DOI:** 10.1097/MD.0000000000030311

**Published:** 2022-08-26

**Authors:** Guang Yang

**Affiliations:** a Department of Pathology, Okayama University Graduate School of Medicine, Dentistry and Pharmaceutical Sciences, Okayama university, Okayama, Japan; b Department of Pathology, Mudanjiang Medical University, Mudanjiang, China.

**Keywords:** gastric adenocarcinoma of fundic gland, microsatellite instability, mismatch repair deficiency, Wnt/β-catenin signaling pathway

## Abstract

Gastric adenocarcinoma of the fundic gland is a rare, well-differentiated variant of gastric adenocarcinoma, which has been proposed as a novel disease entity. As a result of mismatch repair deficiency, microsatellite instability has been frequently observed in various human cancers and widely performed in the area of cancer pathogenesis. Herein, we report a case of gastric adenocarcinoma of fundic gland presented with microsatellite instability phenotype.

**Patient concerns:**

A 46-year-old man was referred to our hospital for abdominal distension and pain.

**Diagnosis:**

The patient contained 3 tumor lesions with different degrees of histologic differentiation and microsatellite instability. The lesions were located in the upper third of the stomach. The tumor size was 55 mm. Macroscopically, tumor showed an ulcerative type. In terms of depth of invasion, tumor lesion invaded into subserosa with lymphatic invasion. In addition, this patient did not present *GNAS* mutation but harbored *AXIN2* mutation. By immunohistochemistry, the expression level of β-catenin protein in the nucleus of the carcinoma cells was obviously higher than that in normal nucleus. Compared with microsatellite instability-low lesion, PD-1, PD-L1, and CD8 were positive in the microsatellite instability-high lesions.

**Interventions:**

The patient underwent surgical resection and postoperative chemotherapy.

**Outcomes:**

The patient experienced distant metastasis and died from severe complications after 6 months of treatment.

**Lessons:**

These results suggested that the mutation of Wnt component genes associated with Wnt/β-catenin signaling pathway activation may play a role in promoting the occurrence of gastric adenocarcinoma of fundic gland. This is the first report of a gastric adenocarcinoma of fundic gland with microsatellite instability. These findings modify our understanding of the pathophysiology of gastric adenocarcinoma of fundic gland.

## 1. Introduction

In 2007, a gastric adenocarcinoma with chief cell differentiation was first reported by Tsukamoto.^[1]^ Not long after that, in 2010, the concept of gastric adenocarcinoma of the fundic gland (GA-FG) as a novel tumor entity was proposed by Ueyama.^[2]^ GA-FG is a rare, well-differentiated tumor with good prognosis. However, in this report, the patient showed malignant aggressiveness with distant metastasis. The detailed pathogenesis remains unclear. Although GA-FG is very rare, the number of diagnosed cases has increased slightly due to the increasing awareness of the disease among pathologists.

Microsatellites (MS), also known as short tandem repeats or simple repetitive sequences, are widely distributed in biological genomes, accounting for about 3% of the genome.^[3]^ Mutation or epigenetic change of MMR genes may lead to a functional loss of normal MMR proteins. Therefore, MS are highly variable, and the wrong accumulation of mutations forms the so-called microsatellite instability (MSI) phenotype.^[4]^ Compared with microsatellite stable (MSS) tumors, MSI tumors often have a heavy mutation burden.^[5]^ In the gene coding regions, MSI often leads to the inactivation of some genes function (such as frameshift mutation), resulting in abnormal protein expression, which may be involved in many pathways related to carcinogenesis. MSI has been frequently observed in various human cancers, including colorectal cancer, gastric cancer, and endometrial cancer. MSI analysis has been widely used in the area of carcinogenesis.^[6–8]^ Therefore, MSI may be one of the most significant mechanisms of carcinogenesis.

Here, we aimed to detect the MSI status of GA-FG, the expression of immune checkpoint molecules (PD-1 and PD-L1), and the mutation status of *GNAS* and Wnt/β-catenin signaling pathway related genes *AXIN2* and *CTNNB1*. To the best of our knowledge, this is the first reported case of a GA-FG tumor with MSI and multiple lesions.

## 2. Case report

### 2.1. Methodology

#### 2.1.1. Patients and tissue samples.

This patient diagnosed with multi-lesion GA-FG was from the pathological department of Mudanjiang Medical University Affiliated Hongqi Hospital in China. The tumour and matched normal tissues were separated from serial sections (10 μm), which were obtained from formalin-fixed, paraffin-embedded (FFPE) tissue blocks by using the microdissection technique.

#### 2.1.2. DNA extract.

Tumour tissue and matched normal tissue were digested overnight at 56°C and the DNA was extracted using a Qiagen DNA Extraction Kit by following the manufacturer’s instruction. The concentration of extracted DNA was determined using a NanoDrop spectrophotometer.

#### 2.1.3. MSI analysis.

In this study, Fluorescently-labeled primers were applied to amplify 5 quasi-monomorphic mononucleotide markers (NR-21, NR-22, NR-24, BAT-25, and BAT-26) for MSI assessment by PCR. The forward primer of each biomarker was labeled with fluorescent dye. Detailed conditions and the sequences of each primer pair were available (see Table, Supplemental Content 1, http://links.lww.com/MD/H146 which illustrates the forward and reverse sequences of each primer pair, corresponding annealing temperature, and PCR products size). PCR products were sequenced using an ABI 310 gene sequencer. Finally, Peak scanner software version 1.0 was used to analyze the sequencing data and the data between the lesion and normal tissues were compared.

In 1997, the American National Cancer Institute proposed that MSI-H was defined as the shift of more than or equal to 2 MS loci among the 5 MS markers. Similarly, when only 1 MS marker size changed, it was defined as MSI-L; when no MS marker shift was observed, it was defined as MSS.

#### 2.1.4. IHC for MMR proteins, cell differentiation markers and CD8/PD-1/PD-L1.

The expression of MMR proteins and cell differentiation markers in GA-FG patient was detected by IHC. FFPE tissue sections with a thickness of 4 μm were prepared and subjected to IHC. The slides were dewaxed in xylene and hydrated in gradient alcohol. After antigen retrieval in sodium citrate buffer, sections were incubated overnight at 4°C using monoclonal antibodies against MLH1 (clone G168-15; 1:50; BD Pharmingen, San Diego, CA), MSH2 (clone G219-1129; 1:200; BD Pharmingen), MSH6 (clone 44/MSH6; 1:100; BD Pharmingen), and PMS2 (clone A16-4; 1:200; BD Pharmingen), MUC6 (clone CLH5; 1:100; Novus, America), pepsinogen-I (clone 8003 (99/12); 1:200; Novus Biologicals, USA), MUC5AC (clone CLH2; 1:100; DAKO, Denmark), H+/K+-ATPase (clone 1H9; 1:50; Fitzgerald, MA, USA), CD8 (clone SP57; 1:100; Roche), PD-L1 (clone SP263; rabbit monoclonal primary antiPD-L1 antibody, prediluted, Ventana Medical Systems, Tucson, AZ), and PD-1 (clone ab137132; 1:250; Abcam). For MMR proteins in tumor tissue, as long as the lack of nuclear staining in cancer cells but positive in surrounding stromal cells, tumor cells are scored as negative expression of MMR proteins. The tumor is defined as MMR-proficient if all MMR proteins are expressed while the tumor is defined as dMMR if absence of expression was detected for at least one of the 4 MMR-proteins. At high magnification (400×), the proportion of tumor infiltrating positive lymphocytes was calculated. The percentage of CD8+ tumor infiltrating lymphocytes <10% represented low CD8 + expression, 10–39% represented moderate expression, and 40–90% indicated high expression. For PD-L1, if more than or equal to 1% of tumor cells (membrane and cytoplasmic staining) were stained, this case was then considered to be PD-L1 positive. For PD-1, if more than or equal to 5% of tumor infiltrating lymphocytes were stained, this case was then considered PD-1 positive.

#### 2.1.5. PCR amplification and mutational analysis of CTNNB1, AXIN, and GNAS.

FFPE sections (10-μm thickness) were dewaxed in xylene and hydrated in gradient alcohol. Then they were slightly stained with HE. The tumor tissue area was clearly identified and separated from the section by using a modified microdissection optical microscope. All PCR conditions except annealing temperature were the same as those of MSI markers amplification. Purified PCR products were sequenced with dideoxynucleotides (BigDey Terminator v3.1; Applied Biosystems, Foster City, CA) and specific primers by a capillary sequencing machine (3730xl Genetic Analyzer; Applied Biosystems). Sequencing data was analyzed using chromas software. A mutation is defined if the height of the mutant peak reaches 20% of the normal peak. Mutations were evaluated by 3 independent researchers among the authors.

## 3. Results

### 3.1. Clinicopathological characteristics

The clinicopathological features of the patient are summarized in Table 1. The patient had a multi-lesion GA-FG with MSI, which contained 3 tumor lesions with different degrees of histologic differentiation. The age of the male patient was 46 years. The lesions were located in the upper third of the stomach. The tumor size was 55 mm. Macroscopically, tumor showed an ulcerative type. In terms of depth of invasion, tumor lesion invaded into subserosa with lymphatic invasion. The patient underwent surgical resection and postoperative chemotherapy. Finally, the patient experienced distant metastasis and died from severe complications after 6 months of treatment.

**Table 1:** Clinicopathological characteristics of the patient.

Case	Age (y)	Sex	Location	Size of tumor (mm)	Macroscopic type	Depth of involvement	Ly	v	H. pylori
1	46	M	U	55	Ulcerative, type 2	pT3	+	−	−

U = Upper one third of the stomach, M = Male, v = venous invasion, Ly = lymphatic invasion, H. pylori = Helicobacter pylori infection.

### 3.2. Hematoxylin-eosin staining and IHC for cell differentiation markers of GA-FG

**Figure 1.**
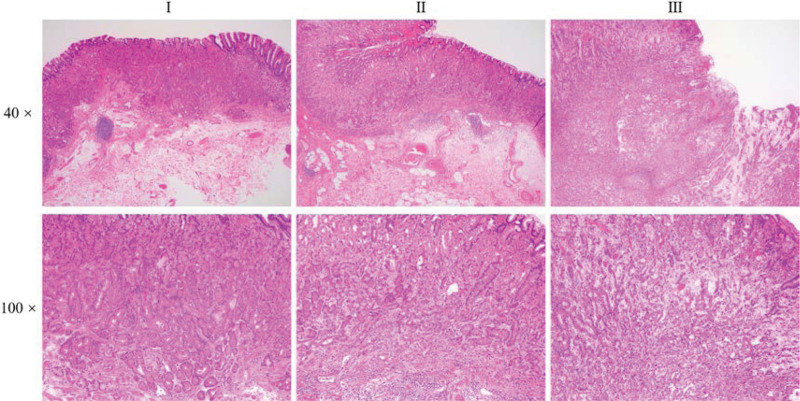
Hematoxylin-eosin staining of patient with 3 divergently differentiated lesions. I, II, and III represented well-, moderately-, and poorly-differentiated GA-FG, respectively. The top and bottom rows represented 40× and 100× magnification, respectively.

Representative pictures are shown in Figure 1. Carcinomatous tissues with 3 different histologic differentiations were observed, named as I, II, and III, which represented well-, moderately-, and poorly-differentiated GA-FG, respectively. Lesion-I presented as well-differentiated and circumscribed tubular adenocarcinoma, which was composed of slightly atypical columnar cells with basophilic cytoplasm and slightly atypical nucleus, mimic to chief cells of fundic gland. The glands were arranged as irregular branching and anastomosing tubes in the lamina propria layer, and sometimes even infiltrated into the submucosa, forming the so-called “endless glands”. The surface of the mucosa was usually covered with nonatypical foveolar epithelium.

**Figure 2.**
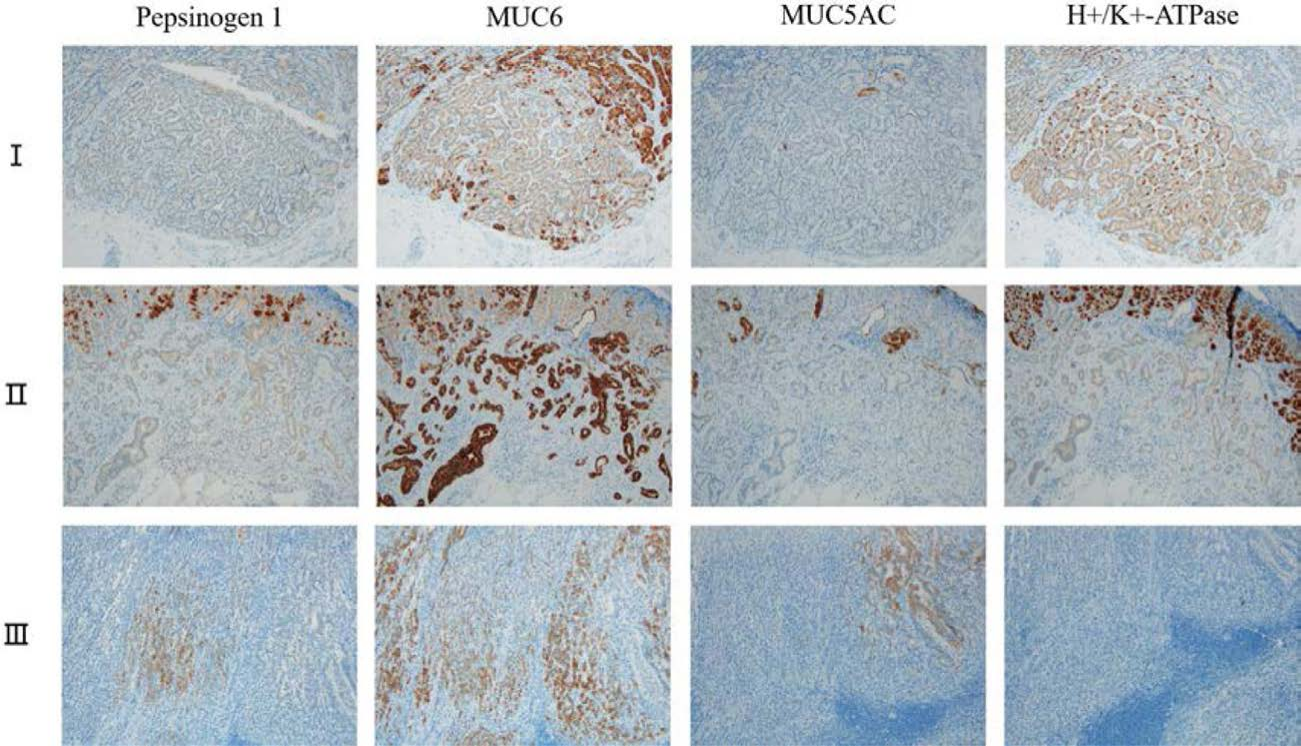
Immunohistochemical staining of cell differentiation markers. The upper, middle, and bottom rows represented lesion I, II, and III, respectively. From left to right, it showed expression of pepsinogen-I, MUC6, MUC5AC, and H+/K+-ATPase. Lesion I: scatteredly positive for MUC6 and H+/K+-ATPase; lesion II: diffusely positive for MUC6; lesion III: focally positive for pepsinogen-I and MUC6.

The results of immunostained cell differentiation markers are presented in Figure 2. All lesions diffusely or scatteredly or focally expressed pepsinogen-I or MUC6 or H+/K+-ATPase. Based on other relevant study, fundic gland cells of GA-FG patients variably express the following biomarkers: MUC6 for mucous neck cells; H+/K+-ATPase for parietal cells; and pepsinogen-I for chief cells.^[9]^ Therefore, it was diagnosed as GA-FG.

### 3.3. MSI and MMR proteins analysis

**Figure 3.**
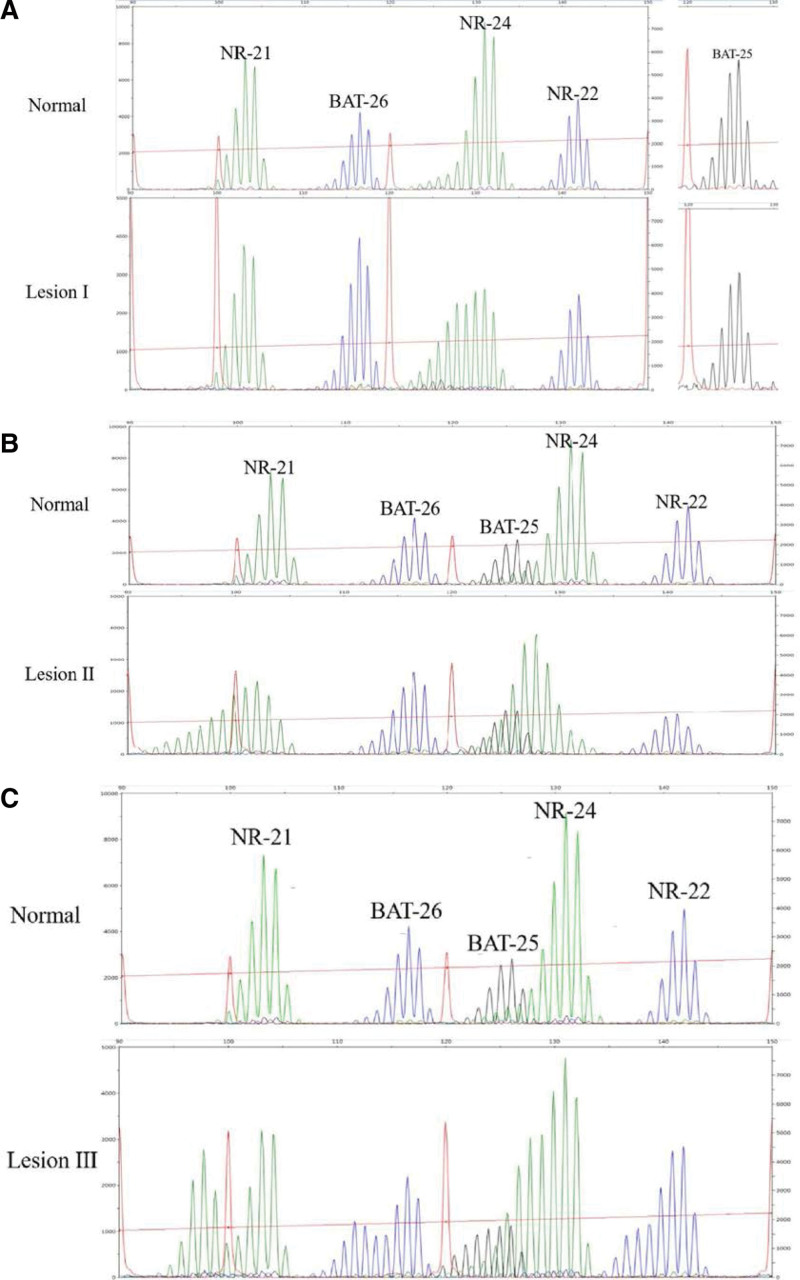
MSI analysis. A, B, and C represented MSI analysis results of lesion I, II, and III, respectively. A, MSI-L; B and C, MSI-H.

We used a panel of quasi-monomorphic mononucleotide repeat markers (NR-21, NR-22, NR-24, BAT-25, and BAT-26) to detect the MS status. We found that the patient contained 3 ectopic lesions that showed different MS phenotype. Compared with the normal tissues, lesion I existing only 1 MS marker shift was defined as MSI-L, and lesion II and III presenting 2 and 4 MS markers shift, respectively, were defined as MSI-H (Fig. 3). Therefore, this patient was identified as MSI-H phenotype by comprehensive evaluation.

MMR proteins loss can be used as an indirect evidence of MSI status. The expression of MMR proteins (MLH1, MSH2, MSH6, and PMS2) were evaluated by IHC. Except for MSH2, the all other 3 proteins were lost in I, II, or III (see Figure, Supplemental Content 2, http://links.lww.com/MD/H147, which illustrates the expression level of MMR proteins in these 3 lesions).

### 3.4. Expression of CD8/PD-1/PD-L1

**Figure 4.**
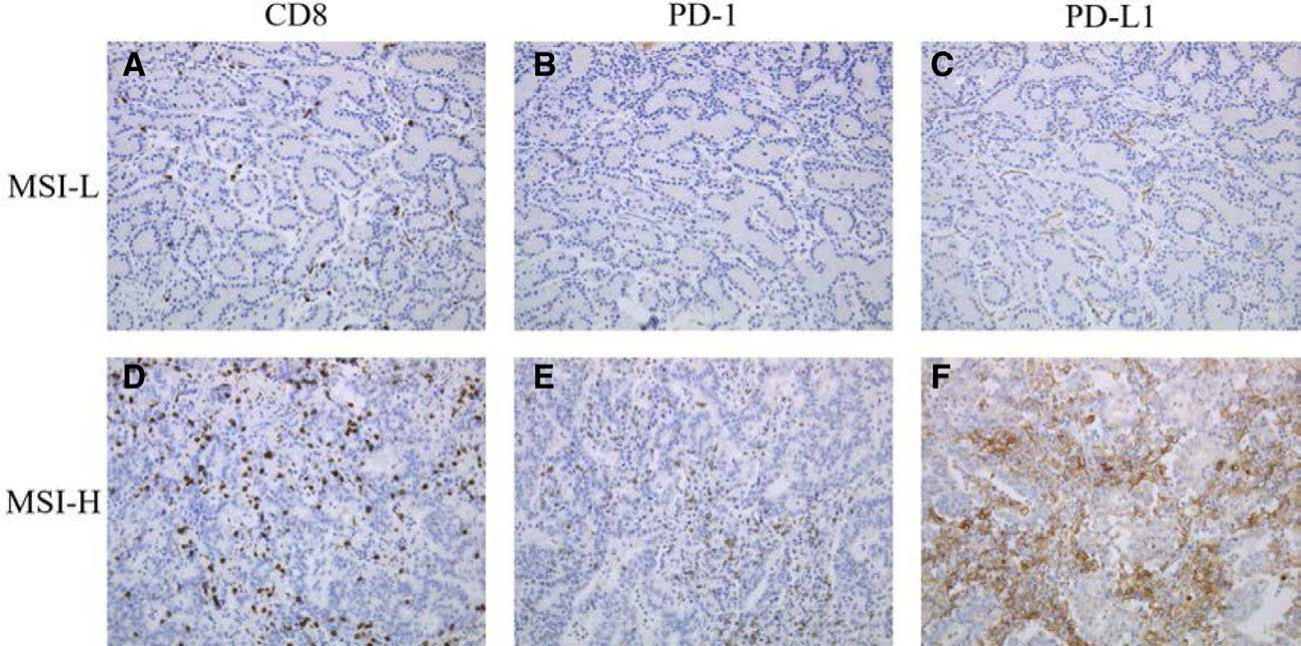
A, D: Immunohistochemical staining of CD8. A, CD8 low expression of CTLs; D, CD8 high expression of CTLs. B, E: Immunohistochemical staining of PD-1. B, no expression of PD-1; D, positive expression of PD-1. C, F: Immunohistochemical staining of PD-L1. C, no expression of PD-L1; F, positive expression of PD-L1.

The expressions of CD8, PD-1, and PD-L1 were detected by immunohistochemistry. In lesion-I, CD8, PD-1, and PD-L1 all were negative, while in lesion with MSI-H, a large number of activated CD8+ CTLs infiltrated into tumor tissue. At the same time, there was high level expression of immune checkpoint molecules (PD-1 and PD-L1) (Fig. 4).

### 3.5. Analysis of GNAS, AXIN, CTNNB1 mutation, and β-catenin expression

**Figure 5.**
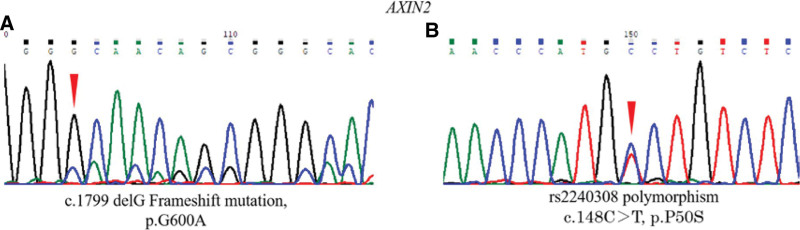
Gene mutation in GA-FG. DelG frameshift mutation (A) and rs2240308 C/T polymorphism (B) at c.1799 and c.148 were observed in tumor-derived DNA, resulting in p.G600A and p.P50S in *AXIN2*, respectively. A: alanine; G: glycine; C: cytosine; T: thymine.

**Figure 6.**
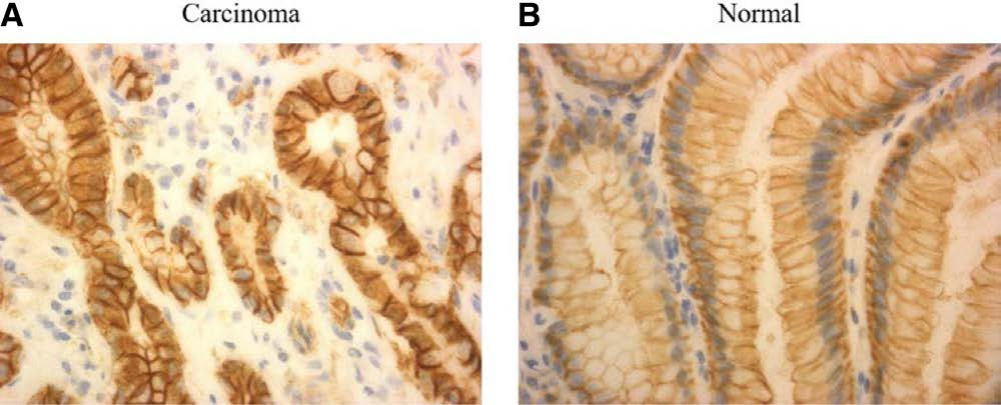
The expression of β-catenin in lesion-II and normal tissue. A, obvious positive staining was observed in nucleus; B, the expression of β-catenin was negative in nucleus and distinct positive in cell membrane in normal tissue.

Tumor tissues were obtained by microdissection. PCR was used for the amplification of *GNAS*, *AXIN*, and *CTNNB1* genes. The DNA sequences were analyzed by capillary electrophoresis sequencing machine, as shown in Figure 5. By sequencing, we found that *AXIN* frameshift mutation (c.1799 del G frameshift mutation) was found only in lesion-II. In addition, *AXIN2* rs2240308 polymorphism (c.148C > T, CCT/TCT, p.P50S) was detected. The detailed mutation data are presented in Table 2. Based on the results of immunohistochemistry, the expression of β-catenin in nucleus was detected between lesion-II and normal tissue. The expression level of β-catenin protein in carcinoma cells was obviously higher than that in normal nucleus (Fig. 6).

**Table 2:** Summary of *GNAS*, *AXIN1*, *AXIN2*, and *CTNNB1* mutations.

			*GNAS*		*AXIN1*		*AXIN2*		*CTNNB*	
Case		MS status	Nucleotide	Amino acid	Nucleotide	Amino acid	Nucleotide	Amino acid	Nucleotide	Amino acid
1	I	MSI-L	-	-	-	-	-		-	-
	II	MSI-H	–	–	–	–	c.1799 delG Frame shift mutation	p.G600A	–	–
	III	MSI-H	–	–	–	–	–	–	–	–

## 4. Discussion

GC is one of the most common malignant tumors in the world.^[10]^ According to relevant studies, the prognosis of GC may not only depend on the stage of the disease, but also be related to the specific molecular biological characteristics of the disease.^[10,11]^ In 2014, the genetic characteristics of GC were described by The Cancer Genome Atlas (TCGA) research network, which confirmed that GC is a complex and heterogeneous disease. Based on the classification of TCGA research work, GC is divided into 4 subtypes: (1) Epstein-Barr virus (EBV)-positive tumors; (2) MSI; (3) Genomically stable (GS); (4) Chromosomal instability (CIN).^[12]^

MSI GC is an independent subtype. According to previous reports, this subtype of GC has positive correlation with female, elderly patients, histologically intestinal type, middle or lower third of gastric position, lower number of lymph-node metastases, and H. pylori infection.^[13]^ GA-FG as a rare, novel, and well-differentiated tumor entity was first reported in 2007 by Tsukamoto^[14]^ and before then, relevant research on MSI GA-FG had not been reported. This may be the first report about multi-lesion GA-FG with MSI. The patient was at an advanced stage and had a larger tumor volume and deeper invasion accompanied with lymph node metastasis, but he showed no H. pylori infection. It has been reported that H. pylori infection can lead to genetic and epigenetic changes through activation of intracellular signaling pathways in gastric epithelial cells, such as hypermethylation of *MLH1* gene promoter and induction of chronic gastric inflammation, atrophic change, intestinal metaplasia, and cancer,^[15–17]^ which present a strong relationship between H. pylori infection and dMMR/MSI cancer. Due to the limited sample size, this MSI GA-FG patient may be an exclusive case.

In this report, we found a rare and multi-lesion GA-FG that was composed of 3 differently differentiated lesions: I, II, and III. I, II, and III were well-differentiated MSI-L, moderately differentiated MSI-H, and poorly differentiated MSI-H GA-FG, respectively. With increased levels of MSI, tumor differentiation also changed from well to poor. It has been reported that compared with solitary tumor patient, MSI-H phenotype is more common in patients with multiple and synchronous GC.^[18,19]^ After resection of early GC, MSI-H phenotype can increase the frequency of synchronous and heterochronous GC occurrence.^[19]^ Although the detailed mechanism of MSI leading to multiple GC is unknown, we believe that this should be related to the heavy mutation burden in MSI-H patients, which promotes the occurrence and development of severe malignancies. Clinically, MSI-H may also be used as a molecular marker for multiple GC prediction. In 2018, Kai K et al reported a case of GA-FG with signet ring cell component.^[9]^ The authors explained their observation with the hypothesis of dedifferentiation of well-differentiated GA-FG into signet ring cells. However, they did not check the MSI phenotype. We speculate that MSI-H status may play a certain role in promoting the transformation of tumor cells from well-differentiation to poor-differentiation.

The expression of CD8, PD-1, and PD-L1 were detected by IHC. We found that the number of infiltrating activated CD8 + CTLs in MSI-H tumor tissue was significantly higher than that in MSI-L lesion, and there was higher level expression of immune checkpoint molecules (PD-1 and PD-L1). MSI-H/MMRD tumor patients have more severe mutation burden (such as frameshift mutation), which often leads to nonself, novel abnormal protein products (such as truncated protein). These abnormal endogenous proteins can induce the activation of CTLs, resulting the infiltration of CTLs into tumor tissue and initiation of tumor-specific immune response.^[20]^ However, interferon γ released by CTLs can induce PD-L1 expression in tumor cells. At the same time, tumor cells drive up-regulation of PD-1 expression on CTLs through transcellular kynurenine (Kyn)-aryl hydrocarbon receptor (AhR) pathway.^[21]^ PD-1 and PD-L1 are negative immunoregulatory factors. The combination of PD-L1 on the surface of tumor cells and PD-1 on the surface of CTLs can inhibit the specific antitumor immune response of CTLs to achieve the immune escape of tumor cells. PD-1/PD-L1 pathway can be blocked pharmacologically through the use of immune checkpoint inhibitors to obtain antitumor immune response again. Therefore, MSI-H phenotype can be used as a predictive marker to screen GA-FG for immunotherapy, and advanced GA-FG patients with MSI-H may benefit from immunotherapy.

Next, we performed genes profiling on this GA-FG patient. For *GNAS* gene, previous research reported that 5 of 26 GA-FG cases contained activated *GNAS* mutation. In their study, *GNAS* mutation seemed to be associated with deeper submucosal infiltration and increased tumor sizes.^[22]^ However, in our study, no activated *GNAS* mutation was found. We speculate that the discrepancy may be attributed to our small sample size or ethnic differences.

In humans, Wnt/β-catenin signaling pathway is one of the most important intercellular signaling pathways. It plays an intrinsic role in cell proliferation, differentiation, regeneration, and some other cellular functions.^[23]^ When cells receive Wnt signal, β-catenin protein will be stably accumulated in the cytoplasm, transferred to the nucleus, and then regulate the expression of downstream target genes; without Wnt protein stimulation, β-catenin protein will be degraded by proteasome through activated multiple proteins degradation complex.^[24]^ Mutation of related genes in the Wnt/β-catenin signaling pathway will lead to abnormal regulation of Wnt/β-catenin signaling pathway. AXIN2, a signal transduction inhibitor and scaffolding protein of Wnt/β-catenin signaling pathway, in combination with APC, GSK3, β-catenin, and DVL, form β-catenin degradation complex. Mutation of *AXIN2* will lead to invalid block of Wnt/β-catenin signaling pathway, which has been reported to be related to the occurrence of various tumors.^[24–27]^ Therefore, we selected *AXIN1*, *AXIN2*, and *CTNNB1* involved in this pathway as target genes for analysis. We observed a frameshift mutation in *AXIN2* in this MSI patient (c.1799 del G frameshift mutation). Under this circumstance, *AXIN2* can encode more frameshift mutant peptides, and the structure and function of AXIN2 protein may be completely changed. Further, it will result in continuous activation of the Wnt/β-catenin signaling pathway.^[28]^ The functional domains of AXIN2 protein include RGS domain (AA: 81–200), which acts as an APC binding site. The binding of AXIN2 and APC is considered to be key to the regulation of β-Catenin level. The mutation of *AXIN2* or *APC* gene may change the conformation of the binding site of the 2 proteins, resulting in a decreased affinity between the 2 proteins and the occurrence of cancer. Therefore, the RGS domain of *AXIN2* acts as a tumor suppressor gene.^[29]^ *AXIN2* rs2240308, a substitution of nucleotide C by T, results in amino acid alteration from P to S at codon 50. Although the single nucleotide polymorphism (SNP) of *AXIN2* rs2240308 at codon 50 is not located in the APC binding site, the site of codon 50 is quite close to the RGS domain, which may affect the affinity between AXIN2 and APC, leading to abnormal regulation of β-Catenin and permit carcinogenesis,^[28]^ which have been reported in lung and prostate cancer. In our study, *AXIN2* rs2240308 SNP was also observed.

In conclusion, the present study confirmed that mutations of genes involved in the abnormal activation of Wnt/β-Catenin signaling pathway were observed in GA-FG, and the abnormal regulation of Wnt/β-Catenin signaling pathway may partly be associated with the occurrence of tumor. Moreover, MSI/MMRD can induce the development of multiple GA-FG, and dysfunction of MMR system may play a key role in tumor occurrence and progression. MSI phenotype can be used as a predictor for immunotherapy for advanced GA-FG. Therefore, clinically, the combined use of immune checkpoint inhibitors and Wnt/β-Catenin signaling pathway blockers may be effective therapy targeting GA-FG.

## Author contributions

All work involved in this study was finished by Guang Yang.

## Supplementary Material


