# Supplementary material for: Microsatellite instability/mismatch repair deficiency and activation of the Wnt/β-catenin signaling pathway in gastric adenocarcinoma of the fundic gland: A case report
Source: Medicine (Baltimore). 2022 Aug 26;101(34):e30311. doi: 10.1097/MD.0000000000030311 (PMC9410697; doi:10.1097/MD.0000000000030311)
Supplement: Supplementary file 1 [file medi-101-e30311-s001.pdf]

Primer	Sequence	Product size （bp）	PCR cycle	AT
GNAS exon8	F: 5’-GGCTTTGGTGAGATCCATTGAC-3’<br>R: 5’-TGGCTTACTGGAAGTTGACTTTG-3’	110	40	55℃
GNAS exon9	F: 5’-GACATTCACCCCAGTCCCTCTGG-3’<br>R: 5’-GAACAGCCAAGCCCACAGCA-3’	130	40	65℃
NR-21	F: 5’-TAAATGTATGTCTCCCCTGG-3’-FAM<br>R: 5’-ATTCCTACTCCGCATTCACA-3’	103	40	55℃
NR-22	F: 5’-GAGGCTTGTCAAGGACATAA-3’-FAM<br>R: 5’-AATTCGGATGCCATCCAGTT-3’	142	40	55℃
NR-24	F: 5’-CCATTGCTGGAATTTTACCTC-3’-HEX<br>R: 5’-ATTGTGCCATTGCATTCCAA-3’	132	40	55℃
BAT-25	F: 5’-TCGCCTCCAAGAATGTAAGT-3’-TAMRA<br>R: 5’-TCTGCATTTTAACTATGGCTC-3’	124	40	55℃
BAT-26	F: 5’-TGACTACTTTTGACTTCAGCC-3’-FAM<br>R: 5’-AACCATTCAACATTTTTAACCC-3’	120	40	55℃
CTNNB1 exon3	F: 5’-CCAATCTACTAATGCTAATACTG-3’<br>R: 5’-CTGCATTCTGACTTTCAGTAAGG-3’	298	40	50℃
AXIN1 exon5	F: 5’-AAGCACGTACCCAAGTCAGG-3’<br>R:5’-CTGAGTAGCCTCGGGACCTT-3’	196	40	67℃
AXIN2 exon1-1	F: 5’-CCAGACTCAGTGGGAAGAGC-3’<br>R: 5’-CTCCCCCAACCCATCTTC-3’	321	40	55℃
AXIN2 exon1-2	F: 5’-TGTTCCGAACTTTCCTGGAG-3’<br>R: 5’-TGCTTCTTGATGCCATCTCTT-3’	211	40	52℃
AXIN2 exon5	F: 5’-AAGACCCGCAGACGATACTG-3’	244	40	52℃

	R: 5’-ACATGCTTCGTCGTCTGCTT-3’			
AXIN2 exon7	F: 5’-AACCCAGTTTCTTTCCTTCT-3’<br>R: 5’-ATCCCTGCCTCAACCTA-3’	251	40	54℃

Abbreviation: F: Forward; R: Reverse.
