# Supplementary material for: Microsatellite instability/mismatch repair deficiency and activation of the Wnt/β-catenin signaling pathway in gastric adenocarcinoma of the fundic gland: A case report
Source: Medicine (Baltimore). 2022 Aug 26;101(34):e30311. doi: 10.1097/MD.0000000000030311 (PMC9410697; doi:10.1097/MD.0000000000030311)
Supplement: Supplementary file 2 [file medi-101-e30311-s002.pdf]

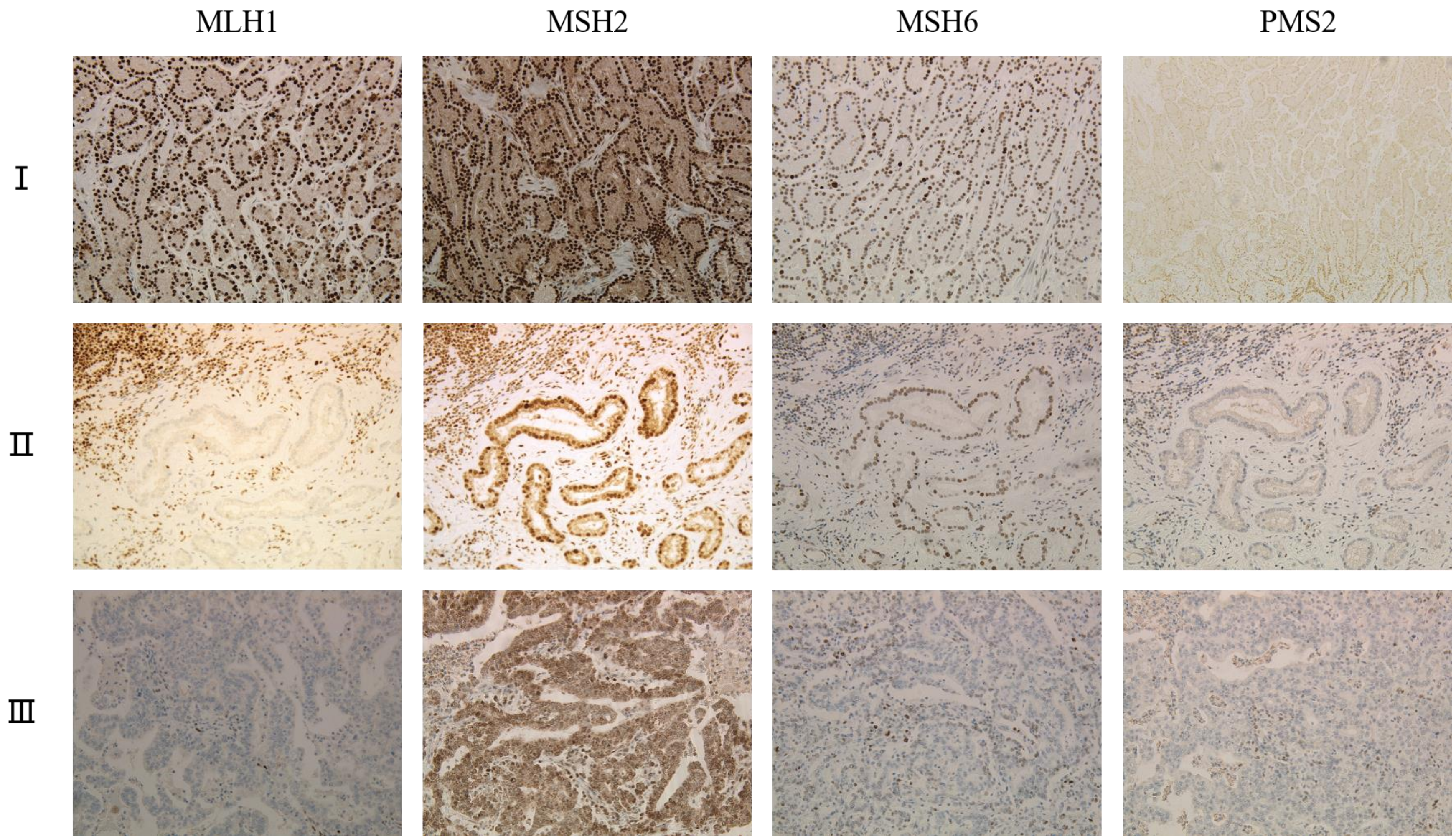


Supplemental Digital Content. The upper, middle and bottom rows represented lesion I, II and III, respectively. From left to right, it showed

expression of MMR proteins MLH1, MSH2, MSH6 and PMS2. For lesion II, MLH1 and PMS2 were both negative; For lesion III, MLH1, MSH6 and PMS2 were all negative.
